# Body Size Mediated Coexistence in Swans

**DOI:** 10.1155/2014/643694

**Published:** 2014-02-04

**Authors:** Katharina A. M. Engelhardt, Mark E. Ritchie, James A. Powell

**Affiliations:** ^1^Appalachian Laboratory, University of Maryland Center for Environmental Science, Frostburg, MD 21532-2307, USA; ^2^Department of Biology, Syracuse University, Syracuse, NY 13244-1270, USA; ^3^Department of Mathematics and Statistics, Utah State University, Logan, UT 84322-3900, USA

## Abstract

Differences in body sizes may create a trade-off between foraging efficiency (foraging gains/costs) and access to resources. Such a trade-off provides a potential mechanism for ecologically similar species to coexist on one resource. We explored this hypothesis for tundra (*Cygnus columbianus*) and trumpeter swans (*Cygnus buccinator*), a federally protected species, feeding solely on sago pondweed (*Stuckenia pectinata*) tubers during fall staging and wintering in northern Utah. Foraging efficiency was higher for tundra swans because this species experienced lower foraging and metabolic costs relative to foraging gains; however, trumpeter swans (a) had longer necks and therefore had access to exclusive resources buried deep in wetland sediments and (b) were more aggressive and could therefore displace tundra swans from lucrative foraging locations. We conclude that body size differentiation is an important feature of coexistence among ecologically similar species feeding on one resource. In situations where resources are limiting and competition for resources is strong, conservation managers will need to consider the trade-off between foraging efficiency and access to resources to ensure ecologically similar species can coexist on a shared resource.

## 1. Introduction

Ecological theory predicts that the number of coexisting species within a community should be finite and that species differ in their morphological traits more than what would be expected by chance [1–4]. Patterns of size differentiation have been observed among various guilds and communities [5–7] and across ecosystems [3, 8], leading to the hypothesis that size differences drive functional differences among species [9] and, therefore, resource partitioning in space [5, 10, 11]. Resource partitioning occurs by eating different types of foods or different size classes of the same food [2, 12–15], where coexistence is possible when species are limited by the resources they can exploit best [16–18]. Much less is known about how body size differentiation may lead to coexistence in the absence of resource partitioning [14]. We illustrate this idea by examining a system featuring ecologically similar swan species feeding on the same resource in space and time. One species, the tundra swan (*Cygnus columbianus*), is common whereas the other species, the trumpeter swan (*Cygnus buccinator*), is rare and federally protected in the United States. Understanding whether and how the two species can coexist on a shared resource is important to the management and conservation of this group of species when food resources are limited.

Two species can coexist on a shared resource when a body size mediated trade-off between foraging efficiency and access to resources exists. (1) Foraging efficiency is defined here as the ratio between foraging gains and foraging costs [19]. Larger-bodied animals incur greater foraging costs when they require more energy for metabolism and locomotion than their smaller-bodied counterparts [3, 20, 21]. However, larger animals gain more energy per unit foraging time because they are more effective in searching, handling, and processing their prey [20, 22–24]. Thus, how foraging efficiency is related to body size depends on body size mediated foraging gains relative to foraging costs. (2) Similarly, access to resources can be mediated by body size through physical or behavioral mechanisms. Longer necks and bodies allow larger-bodied animals physical access to exclusive resources, such that smaller species are included entirely within the niche of the larger species [3, 14, 25]. Likewise, larger animals are generally more likely to displace smaller-bodied individuals through aggressive behavior [21, 26–31].

If larger species indeed have greater access to resources, then species coexistence may be mediated by body size differentiation if smaller species are more efficient foragers. For example, the niche of one species may be entirely included within the niche of a second species. For species to coexist under these conditions [32, 33], the species with the narrower included niche must be more efficient in exploiting the shared resources, and the species with the broader niche must not achieve sufficient density on exclusive resources to numerically outcompete the included niche species for the shared resources (“included niche” hypothesis) [34–36]. Similar to the included niche scenario, two species can coexist when the larger species can displace the smaller species from lucrative foraging locations through aggressive behavior, but the smaller species is more efficient in exploiting the shared resources (“shared preference” hypothesis) [37–39].

Tundra and trumpeter swans are often observed sharing the same habitats in space and time [40] and feed on the same resource, sago pondweed (*Potamogeton pectinatus*) tubers, while staging and wintering in Utah because the tubers are the only available food source during that time [41–43]. We explored the hypothesis that differences between swan body sizes allow the two species to coexist on one resource via an included niche. For the included niche hypothesis not to be rejected, tundra swans would have to be more efficient foragers, but trumpeter swans would have to have access to tuber resources buried deep in wetland sediments. We also tested the shared preference hypothesis that trumpeter swans are superior competitors for the food resources through interspecific aggression. If so, the two species can coexist even without access to exclusive resources if a trade-off exists between superior foraging efficiency of tundra swans and aggressive behavior by trumpeter swans.

## 2. Methods

To assess the potential for tundra and trumpeter swan coexistence via body size mediated trade-offs, we sought to estimate foraging efficiency of the two species versus either niche breadth (amount of shared and exclusive resources available to the swans, included niche hypothesis) or aggressive behavior (shared preference hypothesis). Because assessment of energy gains and costs associated with body size is inherently difficult and would have required extensive handling and undue stress on a federally protected species, we estimated foraging efficiency using a mechanical estimation approach. By measuring the quantity and distribution of food resources in wetland sediments, we could calculate the amount of shared and exclusive tuber resources available in wetland sediments. Behavioral observations documented interspecific aggression and displacement of individuals from foraging locations.

### 2.1. Species and Study Area

We studied tundra and trumpeter swans and their food resources at the Bear River Migratory Bird Refuge (BRMBR divided into 3 separate wetland units; Unit 1, Unit 2, and Unit 4) and the Bear River Club Company (BRC) in northern Utah (41°27′N, 112°18′W). Trumpeter swans are rare in Utah; hence, 57 trumpeter swans from Idaho were released by state (Utah Division of Wildlife Resources, Idaho Department of Fish and Game) and federal (U.S. Fish and Wildlife Service) agencies into BRC in November and December 1996 to expand their wintering range [43, 44]. Tundra swans are abundant throughout the study area during the fall, winter, and spring months (as estimated by the Utah Division of Wildlife Resources every two weeks in October, every week in November and December, and once in January and March with surveys of low-flying aircraft).

### 2.2. Tuber Resources

We quantified biomass of sago pondweed tubers before swans migrated through or wintered on the study areas in the four study wetlands. We resampled the same areas in March after the swans had left to migrate north to their breeding grounds. We placed 64 transects in the 4 wetlands using a two-stage systematic-random sampling design, where each transect was randomly placed within a 1 km^2^ area, and each 1 km^2^ area was part of a grid covering the 4 study wetlands. Transects were 200 m long with one core taken every 20 m. Cores (5.2 cm diameter) were between 15 cm and 45 cm long depending on the depth of the Calcium hardpan that delineates the depth to which sago pondweed tubers are produced. We divided cores into 5 cm sections to explore the 3-dimensional distribution of tuber biomass in wetland sediments, which allowed us to calculate the shared and exclusive portions of the tuber resource. We washed all samples through a sieve with 1 mm openings to extract all tubers, which we subsequently dried and weighed.

To test the hypothesis that swans have access to bigger tubers found in deeper sediments [45, 46] and preferentially feed on bigger tubers, we first determined the relationship between tuber length and depth in Utah sediments and then compared tuber length distributions in wetland sediments to tuber length distributions in swan esophagi. We determined the diets of tundra swans by extracting the gizzards and esophagi of 50 swans killed by swan hunters. We then measured the length and mass of all tubers found in the gizzards and esophagi. Trumpeter swan diets could not be determined because this species is protected from hunting. We supplemented the diet information by observing feeding trumpeter and tundra swans during the day (we dyed trumpeter swans pink under their left wing to enhance identification without drawing undue attention to the swans) and locating them by radiotelemetry (Holohill, Canada) at night.

### 2.3. Swan Morphology and Behavior

We reviewed morphological measurements in the literature [47, 48] and measured neck length to estimate the maximum depth each species can potentially forage to by stretching its neck down through the water and sediment. Swans also “tip up” to reach deeper resources; the legs, latter part of the body, and tail are the only body parts remaining above the water surface [49]. Thus, we estimated the maximum reach of a swan as 1.5 times the neck length, corresponding to conservative estimates derived from Owen and Cadbury [50].

We observed mixed tundra and trumpeter swan flocks while they were feeding to test for interactions that result in displacement of a swan from a feeding “hole.” Mixed flocks were observed for 23.5 h across five consecutive days. Observations were made between dawn and dusk when we could locate mixed flocks foraging close to shore (<100 m) in areas that were not frozen shut. Because trumpeter swans were almost exclusively foraging in BRC, our observations were all made in that wetland unit at 4 different locations, including a spillway, and fringe or interior marsh areas. We observed 7 trumpeter swans (5 adults and 2 juveniles) and 7 tundra swans (6 adults and 1 juvenile). Social class could not be determined for either species; however, the trumpeter swans were generally lone adults and juveniles. Tundra swans were generally present as intact family groups. Less than 5% of all individuals in a foraging area were trumpeter swans. Two juvenile trumpeter swans feeding together with one lone adult tundra swan were the only exception. Because density of trumpeter swans was low, we observed trumpeter swans for 75% of the observation time to increase the chance we would observe interspecific interactions. Sampling was randomized among individuals when sample size allowed. Using focal-individual sampling, we recorded the direction and outcome of the interaction and the type of interaction (bite, chase, threat by extending neck, passive displacement via occupation of space, and hiss). We took care not to disturb the swans while they were foraging and observed the swans from behind cover and through binoculars and spotting scopes.

Likewise, we explored whether the two swan species differed in the effort they put into foraging and in the time they spent foraging for tubers. We observed the two species while they were actively feeding on tubers and timed (a) how long they were paddling and (b) how long their heads were submersed when searching under water for tubers. We observed 19 swans (12 trumpeter swans and 7 tundra swans) that were adults (11 swans) or juveniles (8 swans) and watched each swan from when it started foraging until it stopped foraging and either deliberately swam or flew to a new area. Observation times ranged from 10 min to 150 min (762 min total) and were made in BRC, Unit 2, and Unit 4.

### 2.4. Foraging Gains

**Figure 1:**
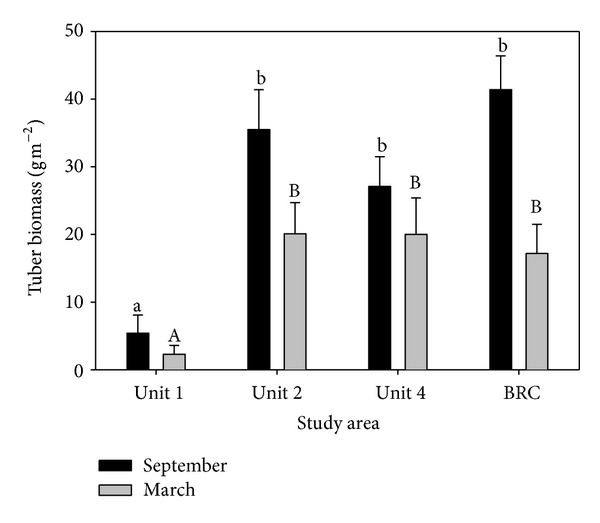
Tuber biomass in sediments of the four study wetlands in September (before swans started foraging) and in March (after swans stopped foraging to migrate to breeding grounds). Multiple Analysis of Variance tested for differences among wetlands and dates. Same letters indicate no significant differences among wetlands. Capitalization emphasizes which observations are compared. Error bars = 1SE.

Nolet et al. [49] estimate tuber harvest rates (*R*_tub_) for tundra swans to be between 0.01 and 0.04 g dry mass s^−1^. We adopt *R*_tub_ = 0.02 g s^−1^ as the average harvest rate in our calculations, which corresponds to a tuber biomass between 20 and 40 g/m^2^ supported by a clayey substrate [49], similar to our estimates of tuber biomass in the study wetlands (Figure 1). If we assume that the digestive processes are not constrained by differences in gizzard size, then foraging gains may be in direct proportion to bill size. Observations on geese show that bite size scales to bill length to power 14.24 and to body mass to power 2.99 [51]. Larger bills of swans may increase encounter rate owing to a larger surface area but larger bills may not be as adroit in extracting tubers from sediments. Because bill length of a trumpeter swan is approximately 1.2 the size of tundra swans [47, 48], we estimate that

(1) RtubTrumpeter=1.2·RtubTundra,

realizing that this may be a liberal estimate and that foraging gains of trumpeter swans relative to Tundra swans may need to be decreased. We keep this uncertainty in estimating resource gain in mind when calculating and interpreting foraging efficiency.

### 2.5. Foraging Cost 

All foraging costs were estimated in J/sec to allow comparisons among estimates. We calculated basal metabolic rate for adult swans using the standard metabolic rate (SMR) equation for homeotherms by Hemmingsen [52] (in Peters [9], page 29):

(2) SMR=4.1W0.751,

where SMR is measured in J/sec and *W* = mass of an individual (10.9 kg for trumpeter swans and 6.8 kg for tundra swans [47, 48]). Kendeigh et al. [53] suggested that the average daily metabolic rate (ADMR) for birds is 1.6 to 2 times SMR in the thermal neutral zone. We use the upper range value to account for extra costs for thermoregulation in the winter. The ADMR estimate includes measurements of existence metabolism (rate of metabolizable energy intake in caged animals maintaining constant body mass outdoors) and estimates of additional metabolic costs of free living. Similarly, Nagy [54] developed allometric equations for mammals and birds to calculate field metabolic rate (FMR). FMR for birds [54] is

(3) FMR=10.9×W0.64.

FMR, similar to ADMR, estimates the costs of free existence. ADMR and FMR were developed for birds during the breeding season and therefore underestimates food requirements and consumption when birds are hyperphagic [54]. Nevertheless, we use ADMR and FMR for both species to obtain an estimate of tundra and trumpeter swan average metabolic energy requirements during one day of free existence keeping in mind that swans may exceed their metabolic requirement during staging or wintering. The estimates of metabolic cost allowed us to calculate the number of swans that the tuber biomass in the study areas could support during staging and wintering.

We identified three foraging costs that could differ substantially among species: (1) cost of flying between foraging locations, (2) cost of swimming between foraging locations, and (3) cost of paddling in one location to stir up top sediment layers [49, 55]. Cost of digging deeper in the sediment layer by bill stabbing and cost of tipping the body vertically to reach further into the sediment are also associated with energetic costs [49]; however, we assumed these costs to be relatively small compared to flying, swimming, and paddling costs and to be similar among the two swan species. Other costs of free existence, such as alertness, posture, reproduction, and growth were also assumed to be either nonexistent at the time of the year, negligible, or similar among the two species. Costs for flying, swimming, and paddling were quantified using mechanistic models because direct measurements on swans were logistically not feasible to obtain. However, we were primarily interested in the ratio of costs between the two species. Potential inaccuracies, which should be similar between the two species, cancel in the ratio.

#### 2.5.1. Costs of Flying

Swans would typically fly from a resting location to a foraging location at dawn and from a foraging location to a resting location at dusk. Swans were also observed flying between foraging locations; however, these foraging flights were relatively infrequent and differences among species could not be observed readily. We predicted short-distance flights to be energetically costly, and energy consumption during lift-off and flight to be dependent on body mass and morphology. We calculated costs of short hop flights for tundra and trumpeter swans [56] using a model for the aerodynamics of bird flight [57]. We estimated parasite power (power required to overcome the drag of the body), induced power (power required to generate lift), and profile power (the power required to overcome the drag of the wings). The total power required for horizontal flight is the sum of parasite, induced, and profile powers, plus an overhead for respiration and metabolism. Parameter estimates and calculations can be found in Powell and Engelhardt [56].

#### 2.5.2. Costs of Swimming 

We calculated the power required for a swan to swim (*P*_swim_) at an average velocity of 0.25 m/sec. A swimming swan is treated as a tugboat [58]:

(4) Pswim=0.011MH2Ovaveswim,

where *M*_H_2_O_ is the mass of the water displaced by the swan, approximately 1/2 of a swan's body mass, and *v*_ave_^swim^ is the velocity at 0.25 m/sec during foraging.

#### 2.5.3. Costs of Paddling

We measured the surface area of the webbed feet and leg length for tundra and trumpeter swans and determined the energy consumed by pressing the areas vertically down a column of water using a fluid mechanics model [59]:

(5) Fpaddle=cDρwaterA2(vavepaddle)2,

where *F*_paddle_ is the force required to move a swan foot through the water column. *ρ*_water_ is 1000 kg/m^3^ at 0–5°C; *A* is the area of foot (141.03 cm^2^ for tundra swans and 167.72 cm^2^ for trumpeter swans); *v*_ave_^paddle^ is the average velocity of a swan foot traveling vertically through the water column, estimated below. *c*_*D*_ is a function of the Reynolds number when assuming that swan feet are circular [59], where Re=ρwatervavepaddleL/μH2O. *c*_*D*_ is constant at 1.01 for *Re* > 3 × 10^3^, which is the case here (*Re* = 3.02 × 10^4^ for tundra swans and *Re* = 3.25 × 10^4^ for trumpeter swans). *L* is the travel length of a swan foot (14.6 cm for tundra swans and 15.7 cm for trumpeter swans traveling at a 30° angle through the water column). *μ*_H_2_O_ = 1.518 × 10^−3^.

The average power requirements for paddling are *P*_paddle_ = *F*_paddle_ *v*_ave_^paddle^. To determine average velocity of paddling, we assumed that the power requirement for paddling vigorously to stir up sediments is the same as that for vigorous swimming, as when swans swim away to avoid observers. This speed is approximately 1 m/sec, a comfortable walking pace over rough ground. We base this estimate on observations of how rapidly we could walk toward swans before they would choose to take flight as opposed to continue swimming away. To determine *v*_ave_^paddle^, we calculated

(6) cDφwaterA2(vavepaddle)3=Ppaddle=Pswim=0.011·MH2O·1 m/sec.

We solved this equation for *v*_ave_^paddle^ and subsequently determined *P*_paddle_ for each species.

### 2.6. Comparative Efficiencies

We define efficiency of each species as the ratio of energy gains to energy expenditures. We calculate a ratio rather than a difference to obtain an efficiency estimate that is dimensionless. In Utah, where the swans do not reproduce and life is reduced to the basics of flying to and from foraging sites, swimming between foraging locations, and foraging for tubers, the daily energy gains and losses can be summarized as

(7) efficiency=foraging  gainsenergy  costs=η(fforageRtubNsec)2·Eflight+Nsec(Pmet+fswimPswim+fpaddlePpaddle),

where *η* is the energy content (joules/gram) of tubers, per dry mass [60]; *f*_forage_ is the fraction of day spent foraging; *R*_tub_ is the rate of tuber intake [49] (grams/second); *N*_sec_ is the number of seconds in one day (seconds); *E*_flight_ is the energy required for one-way flight between resting and foraging sites (joules); *P*_met_ is the standard metabolic rate (joules/second); *f*_swim_ is the fraction of day spent swimming; *P*_swim_ is the power requirement for swimming (joules/second); *f*_paddle_ is the fraction of time spent paddling to uncover tubers;  *P*_paddle_ is the power requirement for paddling (joules/second).

The standard metabolic rate *P*_met_ does not include any costs of free existence, such as costs of swimming (*P*_swim_), costs of paddling (*P*_paddle_), and costs of flight (*E*_flight_). The costs are calculated separately for those activities that we believe may differ substantially among the two species owing to differences in body size. Other costs, such as costs of posture and staying alert, are assumed not to be substantially influenced by body size and are therefore excluded when calculating efficiency. This means that costs are underestimated, but to the same extent for both species. As a comparison, we used FMR as our *P*_met_ estimate to calculate costs that incorporate all costs of free existence.

### 2.7. Statistical Analysis

To estimate shared and exclusive tuber resources, we needed to know the quantity and spatial distribution of tuber biomass in each sampled wetland. We estimated tuber biomass (g/m^2^) for each of the 64 transects sampled and extrapolated the results to the scale of the four wetlands using geostatistical analysis [43]. We quantified exclusive resources by calculating the maximum sediment depth at each transect to which an adult tundra and trumpeter swan might forage after accounting for water depth. We then summed tuber biomass at depths greater than the maximum tundra swan reach but less than the maximum trumpeter swan reach for every wetland. Likewise, we calculated shared resources by subtracting exclusive tuber biomass and tuber biomass not accessible to either species from total tuber biomass.

Swan days are the number of tundra and trumpeter swans that could be supported on a resource for one day (or, how many days one swan could be supported). We estimated tundra and trumpeter swan days for each wetland by dividing tuber biomass of each wetland by the tuber biomass required per individual per day. The daily tuber biomass requirement (DTR) was calculated using two approaches. (1) ADMR (or FMR) for the two species was divided by the metabolizable energy content (dietary energy content of the food minus energy voided in fecal and urinary excretions) of sago pondweed tubers (*η* = 11.7 kJ/g [60]):

(8) DTR=ADMRη.

(2) An allometric equation for birds [54] is

(9) DTR=0.648×W0.651.

We assumed that 25% of tuber biomass was unavailable for consumption owing to decomposition [61]. To understand how limiting tuber resources were, we compared these estimates to estimates of the number of swans frequenting the wetlands between October and March using counts from the 12 aerial flyovers and extrapolating over the entire study period by assuming each count represented the week (November and December), two weeks (October), or the month (January and February) the flyover was conducted. We also compared tuber biomass before swans arrived in September to biomass after swans left in March using Multiple Analysis of Variance (MANOVA) with the two dates and the four wetlands as factors to further explore how much the swans had exploited the tuber resource over the fall and winter period.

We used MANOVA to test the hypothesis that tuber biomass and length increased with sediment depth (5 cm increments corresponding to the 5 cm sediment core sections) and that the relationship would be similar among all sampled wetlands. To test whether swans were preferentially feeding on larger tubers, we developed frequency distributions of tuber lengths collected from swan esophagi and from sediments of the 4 wetlands and then used ANOVA to test for statistical differences in mean tuber length of tuber ingested by swans or found in wetland sediments. Likewise, we compared frequency distributions of tubers collected in September and in March to test whether certain sizes were depleted to a greater extent.

We used a two-population *t*-test to test for differences in neck lengths between the two swan species. To explore differences in foraging behavior, we used MANOVA to test whether species and age influenced the percent time spent paddling while foraging and the time spent foraging for tubers (heads and necks submersed).

All biomass measurements are recorded as 100% dry matter (mean dry mass = 33.34% wet mass). We used SAS software [62] for all statistical tests and S+ for geostatistical interpolation between samples [63]. Unless otherwise noted, we evaluated statistical significance at *P* < 0.05 and we report values as means ± SE.

## 3. Results

### 3.1. Tuber Resources

In September 1996, Unit 1 supported the least average tuber biomass/m^2^ (5 ± 2.7 g/m^2^) followed by Unit 4 (27 ± 4.4 g/m^2^), Unit 2 (35 ± 5.9 g/m^2^), and BRC (41 ± 5.0 g/m^2^). Biomass was significantly different among wetlands (ANOVA; *F*_3,60_ = 7.86, *P* < 0.001) but only because Unit 1 supported significantly lower tuber biomass (Figure 1). In March 1997, 50% of the tuber resources were depleted in Unit 1 (2.5 ± 2.3 g/m^2^), 29% in Unit 4 (20 ± 5.4 g/m^2^), 43% in Unit 2 (20 ± 4.6 g/m^2^), and 59% in BRC (17 ± 4.3 g/m^2^). Comparison of wetlands and dates showed that biomass differed significantly (MANOVA; *F*_4,123_ = 11.73, *P* < 0.001) because Unit 1 produced significantly less tuber biomass than the other wetlands in September and March (Table 1). Tuber depletion in the 4 wetlands reflects swan use during the study period as observed during flyovers, where swans frequented BRC the most (4549 swans per day between October 15 and March 15), Unit 2 supported on average 2821 swans/day, and Unit 4 supported 1854 swans/day. Unit 1 was used almost exclusively as a resting area during the day; hence, counts are inflated and were not used in our analysis as counts of foraging swans. From these counts and accounting for days when the wetlands were frozen and swans could not forage, the wetlands supported 1.5 × 10^6^ swan days between September and March (Table 2). The tuber resources, on the other hand, could potentially support 5.1 × 10^6^ swan days (Table 2). This estimate is calculated by dividing total tuber resources in each wetland, after accounting for 25% tuber biomass loss to decomposition [61], by estimates of daily tuber requirements of both species (see calculations for tuber biomass requirements under Comparative Efficiency). The estimate does not account for swans giving up a foraging area at a tuber biomass or density that is no longer lucrative for maximizing energy intake [64, 65]. It also does not take into account that swans are hyperphagic during staging and will consume more than their daily requirement to gain weight.

BRC was the only wetland that supported exclusive resources during the study period (Table 1). Exclusive tuber resources accounted for 8% of total tuber biomass in BRC or 4% of total biomass in all four wetlands. We estimate that exclusive resources could support 1.7 × 10^5^ trumpeter swan days (Table 2); however, we found no evidence that these resources were actually exploited by trumpeter swans (Table 1). Nevertheless, ground observations and radiotelemetry data suggest that trumpeter swans fed almost exclusively in BRC, corresponding to areas supporting the highest tuber biomass and providing exclusive tuber resources to trumpeter swans.

**Figure 2:**
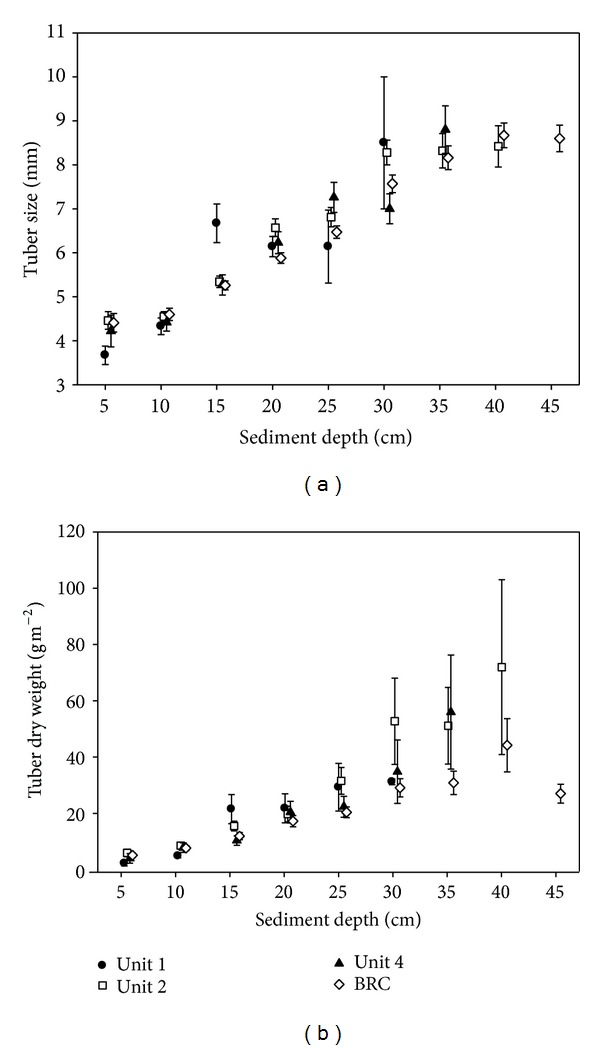
Tuber length (a; mean ± 1SE) and tuber dry mass (b; mean ± 1SE) per 5 cm depth increments (e.g., values at 5 cm depth represent tubers found in 0–5 cm sediment depth) for the four study wetlands. Maximum sediment to a calcium hardpan differed among wetlands, with BRC being the wetland with the deepest sediments.

Tuber length and total biomass increased with sediment depth in all sampled wetlands (Figure 2; MANOVA; *F*_22,732_ = 6.89, *P* < 0.001 for biomass; *F*_22,1122_ = 23.80, *P* < 0.001 for size). Wetlands differed in their mean tuber length and biomass (*P* < 0.01 in both cases). We found a significant interaction (*P* < 0.01) between wetland and sediment depth for tuber length only, which was driven by one of the four wetlands, Unit 1, which supported the least tuber biomass.

**Figure 3:**
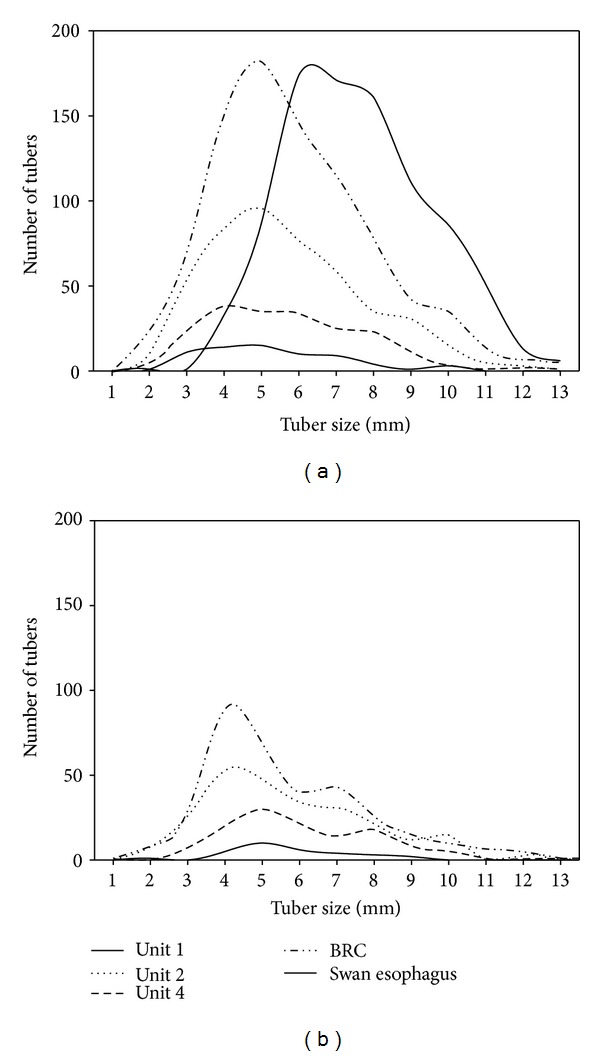
Number of tubers for each tuber length (mm) in a representative swan esophagus (a) and collected from sediment cores of the four studied wetlands in September (a) and March (b). Note that absolute number of tubers is presented to show differences in tuber density among wetlands and seasons. Mean ± 1SE of tuber length for swans: 7.57 ± 0.64 mm, Unit 1: 5.57 ± 0.22 mm, Unit 2: 6.14 ± 0.13 mm, Unit 4: 6.06 ± 0.14 mm, BRC: 6.25 ± 0.07 mm.

Ground and aerial observations indicated that both species exclusively fed in sediment beneath submersed aquatic plant beds, which, in November and December, only provide sago pondweed tubers as a food resource for swans. Of 50 tundra swan gizzards and esophagi collected, 7 contained 100% sago pondweed tubers and no other plant materials. The rest of the esophagi and gizzards were empty. Tuber lengths extracted from tundra swan esophagi were significantly longer than tuber lengths extracted from wetland soils (Figure 3(a); ANOVA; *F*_4,2299_ = 65.52, *P* < 0.001). Frequency distributions of tuber lengths in swan esophagi compared to wetland soils show a mode of 7 mm for swans versus 5 mm for wetland soils (Figure 3(a)). Lengths of tubers sampled from March sediments (Figure 3(b)) show that tubers in Unit 2 (ANOVA; *F*_1,528_ = 6.28, *P* = 0.01) and BRC (ANOVA; *F*_1,1207_ = 18.36, *P* < 0.001) are significantly smaller than tubers collected in September (6.67 ± 0.13 mm in Unit 2 and 5.66 ± 0.12 mm in BRC). Average tuber length for Unit 1 and Unit 4 did not differ between September and March.

### 3.2. Body Size and Behavioral Observations

Body morphology measurements found in the literature [47, 48] and field measurements show that size ratios range from 1.1 to 1.2 for linear estimates (e.g., bill length and neck length) and 1.6 for mass measurements, with trumpeter swans being the bigger of the two species. Trumpeter swans had longer necks (51.04 ± 0.70 cm) than tundra swans (47.05 ± 0.37 cm) (ANOVA; *F*_1,134_ = 31.12, *P* < 0.001).

Behavioral observations showed that swans differed in the percent time they spent paddling while foraging (MANOVA; *F*_2,16_ = 4.71, *P* = 0.02) and the time spent foraging under water (MANOVA; *F*_2,16_ = 9.24, *P* = 0.002). We did not detect significant differences among species. Rather, adults and juveniles exhibited different foraging behaviors, with juveniles spending more time paddling (*P* = 0.07) and less time submersed under water (*P* = 0.001).

Observations on intra- and interspecific interactions among swans showed that out of 43 interactions observed, trumpeter swans displaced tundra swan 26 times whereas one adult tundra swans displaced one juvenile trumpeter swan once during the observation period (Table 3). We observed one juvenile trumpeter swan displacing one adult tundra swan. Field notes suggest that trumpeter swans were clearly the dominant species even when few individuals were foraging in the midst of a large tundra swan flock.

### 3.3. Comparative Efficiency

The calculated standard metabolic rates are 17 J/sec for a 6.75 kg adult tundra swan and 25 J/sec for a 10.86 kg trumpeter swan. Average daily metabolic rate (ADMR) and field metabolic rate (FMR) were both calculated as 35 J/sec for a tundra swan and 49 J/sec for a trumpeter swan. Dividing field metabolic cost by the true metabolizable energy content of sago pondweed tubers (11.7 kJ/g [60]), a tundra swan requires 258.5 g tubers per day (Beekman et al. [61] estimated 283 g per day for tundra swans in the Netherlands), whereas a trumpeter swan requires approximately 361.8 g tubers per day. Using the allometric equation for birds proposed by Nagy [54], the tuber requirement for tundra swans is 201.6 g/day and 274 g/day for trumpeter swans. Because the allometric equation underestimates food consumption during staging [54] and our estimates incorporate the actual energy content of tubers, we used our estimates of the daily tuber biomass requirements while swans stage or winter in Utah to calculate how many swans can be supported on the shared and exclusive portions of tuber resources (see above).

Tundra swans consume approximately 1700 J/sec when flying whereas trumpeter swans consume approximately 3500 J/sec [56]; that is, flight costs are 2.1 times higher for trumpeter swans. Tundra swans consume 0.0375 J/sec while swimming and trumpeter swans consume 0.06 J/sec, that is, swimming costs are 1.6 times greater for trumpeter swans. Likewise, trumpeter swans consume 0.48 J/sec when paddling while tundra swans consume 0.30 J/sec. Thus, paddling is also 1.6 times more costly for trumpeter swans as it is for tundra swans.

Field observations on swans foraging in the BRMBR and BRC wetland units suggest that time spent foraging, approximately 10 h per day (similar to Squires [60]), is roughly equal for each species. Exact foraging time over a 24 h period could not be determined because swans would frequently forage during the night when observations could not be made. However, inaccuracies of foraging time cancel out because we assume that both species forage for approximately the same amount of time. Detailed observations on foraging swans show that, on average, swans spent 10% of their foraging time paddling to uncover tubers and 15% of their foraging time swimming between and searching for lucrative tuber patches. Hence, using values summarized in Table 4 and the energy content of sago pondweed tubers (*η* = 11.7 kJ/g), the efficiency (kJ gain/kJ cost) for tundra swans is 5.2 and 4.2 for trumpeter swans. Gains here greatly exceed costs because not all costs of free existence are accounted for and mechanical costs are not adjusted by an aerobic efficiency. If ADMR or FMR is used rather than SMR for the *P*_met_ estimate, then efficiency decreases to 2.7 for tundra swans and 2.3 for trumpeter swans.

## 4. Discussion

In this study, we explore whether body size differentiation can lead to coexistence even in the absence of resource partitioning when one species incurs lower foraging costs but the other species can gain access to exclusive resources by (a) exploiting the portion of the resource that the other species cannot physically reach (included niche hypothesis) or by (b) displacing the other species from the shared resource through aggressive behavior (shared preference hypothesis). While resources were probably not limiting during the study period, we show that the smaller tundra swan is more efficient in exploiting tuber resources because it incurs lower foraging costs per unit energy gained than the bigger trumpeter swan; however, trumpeter swans have access to exclusive tuber resources that are available to them through their longer necks and bodies (the included niche hypothesis). Even in the absence of exclusive resources, a trade-off exists between lower foraging costs of tundra swans and the aggressive behavior by trumpeter swans that allows them access to lucrative resource locations (the shared preference hypothesis). Thus, in situations where resources are limiting, such as small wetlands with large swan populations, the trade-offs we report may be important in enhancing the coexistence of the two swan species during staging and wintering.

Smaller species are often more efficient foragers [66], where foraging efficiency is defined as the energetic gain per unit cost of foraging [19, 22]. In our case, the smaller tundra swan incurred lower costs associated with flying short distances between foraging and resting areas [56], swimming between tuber patches, and paddling to stir up the water column and sediments. Standard and field metabolic rates are also lower for tundra swans. Thus, extra resource gains to overcome costs need to be at least 1.5 times higher for trumpeter swans. However, trumpeter swans are at most 1.2 times more effective in foraging for tubers if larger bill size allows trumpeter swans to encounter and capture more tubers per unit effort. If not, trumpeter swans are at an even greater disadvantage when comparing energetic gains to energetic costs related to body size. Trumpeter swans have to overcome their greater foraging costs relative to foraging gains by interfering more and/or by feeding longer in one area. Indeed, we found that trumpeter swans were more aggressive (Table 4) and should be less inclined to take off because flights are approximately twice as expensive for trumpeter swans as tundra swans [56]. We did not observe any interspecific differences in time spent foraging in an area. Differences in foraging behaviors need not be large to overcome differences in foraging costs; however, high variance in foraging behavior among individuals of the same species, especially between adults and juveniles, would require the sample size to be unrealistically large to detect differences among species.

Mass intake rate is a function of tuber density, tuber length, and burial depth where larger tubers buried deeper in the sediments may not necessarily yield higher mass intake rates [67, 68]. Mass intake rates are hard to predict without direct experiments. Nevertheless, tuber lengths found in tundra swan esophagi were on average bigger than tubers found in wetland sediments (Figure 3(a)), and longer tubers were depleted to a greater extent in the two study wetlands that most swans foraged in (Unit 2 and BRC; Figure 3(b)). Thus, we can conclude that tundra swans selected bigger tubers that are found deeper in the sediments (Figure 2) where tuber density is lower (compare Figure 2(a) and Figure 3(a)). We have no reason to believe that trumpeter swans would select tubers differently.

Swans left the study area in the spring when the three most productive wetlands reached a surprisingly constant tuber biomass of 17–20 g/m^2^ and a tuber density of 634–672 tubers/m^2^. Wetlands with the highest tuber biomass and density were depleted to a greater extent than less lucrative wetlands (Figure 1), indicating that the swans maximized their energy intake by foraging in the most lucrative areas. Aerial and ground observations, that monitored presence and density of tundra and trumpeter swans through time, corroborate this conclusion. From the tuber depletion measurements and the interaction observations we can conclude that swans were competing for access to the most lucrative tuber patches even though total food resources may not have been limiting. We also conclude that trumpeter swans cannot achieve sufficient density on exclusive tubers alone (Table 2) to outcompete tundra swans for the shared tubers; hence, an included niche scenario is a plausible explanation for species coexistence in our model system.

Even when exclusive resources are absent or cannot be used profitably (no depletion of exclusive resources was detected; Table 1), trumpeter swans, through their aggressive behavior, could gain easy access to shared resources. This scenario is similar to the shared preference/interference system developed and tested by Pimm et al. [37]. In this system, hummingbirds share a preference for a habitat, but, at a high enough density of the dominant species, the subdominant switches to the less preferable habitat. Habitats in our swan system are dominated by the same food item, but density and biomass of the tuber resource differ within and among wetlands. Hence, we predict that trumpeter swans will displace tundra swans from lucrative areas when trumpeter swan densities and harassment pressures are large enough for tundra swans to move away. A trade-off, then, between aggressive behavior of the bigger species and the lower foraging costs of the smaller species may lead to coexistence of the two species [24]. At what trumpeter swan abundance this switch will occur is unknown; during the study, only 57 translocated trumpeter swans were present in the study area and we observed both species foraging in mixed flocks.

In summary, we show that the larger body size of trumpeter swans may indeed allow this species greater access to exclusive and shared tuber resources because of their greater niche breadth and aggressive behavior; however, we found that the larger body size is associated with higher energetic costs. Tundra swans, on the other hand, are able to exploit the shared resources more efficiently. These observations suggest that the two species can coexist even in the absence of resource partitioning when resources are limiting owing to a body size mediated trade-off between greater foraging efficiency by the smaller-bodied tundra swan and greater access to resources by the bigger trumpeter swan.

## 5. Conservation Implications

Our findings suggest that competition with tundra swans most likely cannot account for the absence of trumpeter swans in Utah, even when tuber resources are limiting, because neither species exerts a strong enough negative effect on the other that would lead to a population decline. Thus, other factors, such as differential mortality and nesting success have to account for the absence of trumpeter swans in Utah wetlands. The findings may be applied to other systems, such as the Chesapeake Bay, where managers are concerned about the direct and indirect effects of nonnative mute swans (*Cygnus olor*) on the survival of native swans. In the Chesapeake Bay wetlands, unlike the Utah wetlands, limiting food resources owing to habitat degradation, overly aggressive behavior of mute swans, and a large and nonmigratory population of mute swans may exert a strong negative effect on native swans that may ultimately lead to their decline.

## Figures and Tables

**Table 1:** Amount of total and exclusive tuber resources and the amount of tuber biomass ingested for each wetland between September and March. Exclusive resources are only available to trumpeter swans. All values reported as 10^3^.

Site	Shared kg before	Exclusive kg before	Shared kg after	Exclusive kg after	Shared kg ingested^a^	Exclusive kg ingested^a^
Unit 1	65	0	33	0	16	0
Unit 2	571	0	311	0	117	0
Unit 4	243	0	172	0	10	0
BRC	892	80	340	61	329	0

^a^Accounts for 25% tuber biomass lost to decomposition [59].

**Table 2:** Comparison of tundra and trumpeter swan days that the tuber resource (Table 1) could potentially support and the number of tundra and trumpeter swans actually observed foraging in the four study wetlands. We assume tundra swans require 258.5 g tubers/day (twice basal metabolic rate) and trumpeter swans require 361.8 g tubers/day. Swans are likely to ingest more tuber biomass per day during staging to maximize energy intake.

Site	Calculated tundra swan days on shared resources^a^	Actual tundra swan days^b^	Calculated trumpeter swan days on exclusive resources^a^	Actual trumpeter swan days^b^
Unit 1	189,000	90,000	0	0
Unit 2	1,657,000	418,000	0	0
Unit 4	705,000	127,000	0	0
BRC	2,588,000	821,000	166,000	600

Total	5,138,000	1,456,000	165,000	600

^a^Calculation of swan days does not take into account that swans will not completely deplete the tuber resources in the sediments. Calculations account for 25% tubers lost to decomposition during dormancy.

^b^Calculation of actual swan days takes into account days that the wetlands were frozen shut and swans were not foraging.

**Table 3:** Inter- and intraspecific aggression between and among tundra and trumpeter swans while feeding on sago pondweed tubers. Swans were observed feeding in mixed flocks for 23.5 h. Interactions always resulted in the target individual moving away. Swans actively displaced each other through bites and chases and often threatened with outstretched necks or a hiss. Occasionally, they would passively displace each other by passively occupying the space of a foraging swan (“spatial”). A > B denotes that A wins in an aggressive encounter.

Interaction	Tundra > tundra	Tundra > trumpeter	Trumpeter > tundra	Trumpeter > trumpeter
Bite	3	0	16	3
Chase	5	0	0	1
Neck	2	0	2	0
Hiss	2	0	7	0
Spatial	0	1	1	0

**Table 4:** Calculation of efficiency indices for tundra and trumpeter swans. The index is defined as the ratio between foraging gains and total energy requirements in one day, including costs of free living and mechanical costs of foraging flights, swimming, and paddling. Intake rate for tundra swans (*R*_tub_) was derived from Nolet et al. [49] for clayey substrate. Fraction of each day spent foraging (*f*_forage_), swimming (*f*_swim_), and paddling (*f*_paddle_) is based on observations of foraging swans. Powell and Engelhardt [56] reported *E*_flight_ for a 5 kilometer foraging flight. Mechanical costs are not adjusted by aerobic efficiency.

	Tundra swan	Trumpeter swan
*f*_forage_	0.42	0.42
*R*_tub_ (g/sec)	0.020	0.024
*N*_sec_ (sec)	86400	86400
*E*_flight_ (kJ)	80	120
*P*_met_ (J/sec)	34.60	49.31
*f*_swim_	0.094	0.094
*P*_swim_ (J/sec)	0.0375	0.0600
*f*_paddle_	0.019	0.030
*P*_paddle_ (J/sec)	0.30	0.48
Gain (kJ)	8,424	10,109
Cost (kJ)	3,102	4,432
Efficiency	**2.72**	**2.28**
